# Supplementary figures and images for: Systematic examination of a heart failure risk prediction tool: The pooled cohort equations to prevent heart failure
Source: PLoS One. 2020 Nov 3;15(11):e0240567. doi: 10.1371/journal.pone.0240567 (PMC7608925; doi:10.1371/journal.pone.0240567)

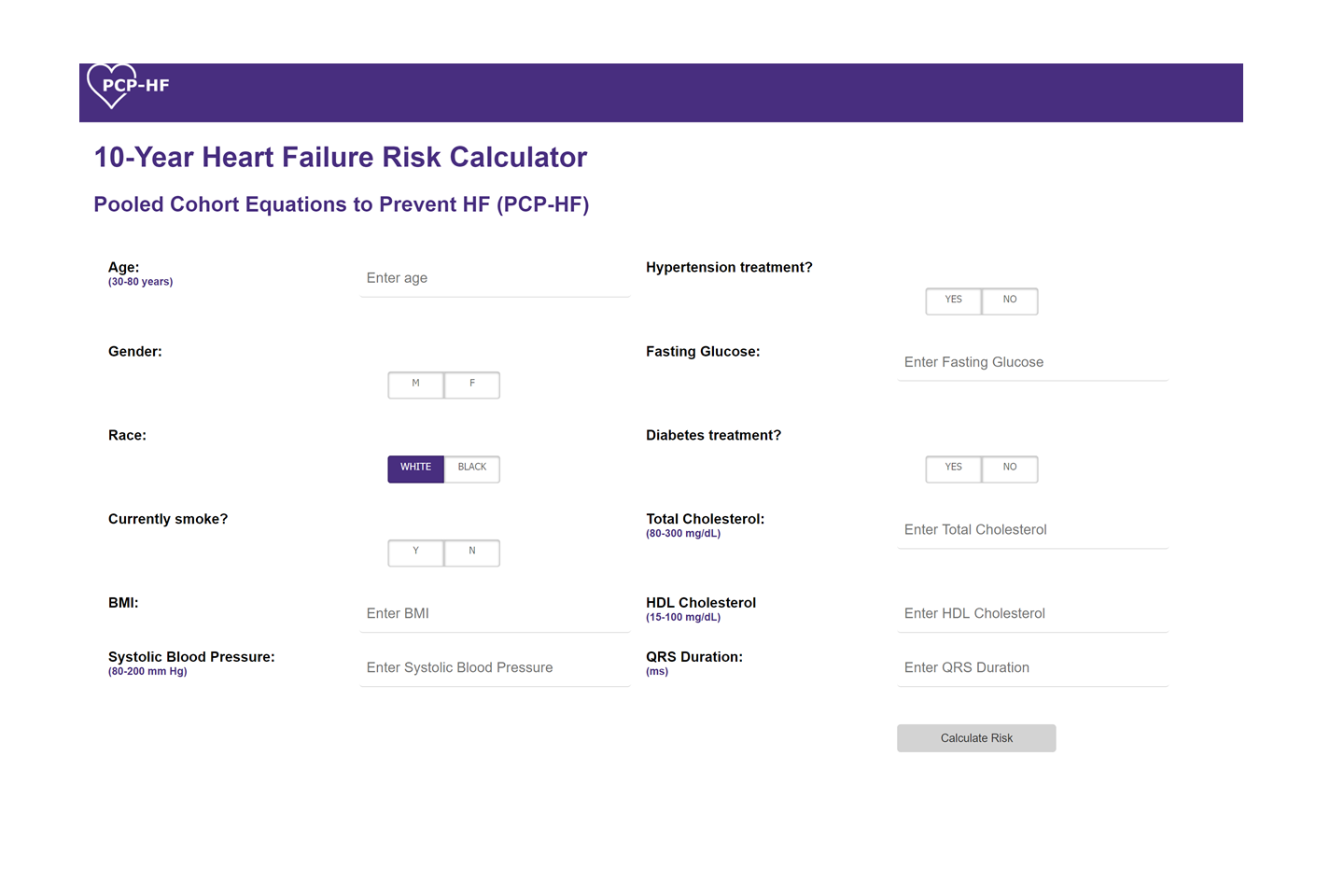

Supplement: S1 Fig — Data routinely available in ambulatory setting can be entered at www.pcphf.org to calculate race-sex specific ten-year risk of incident HF. BMI = body mass index; HDL = high-density lipoprotein cholesterol. (TIF) [file pone.0240567.s001.tif]

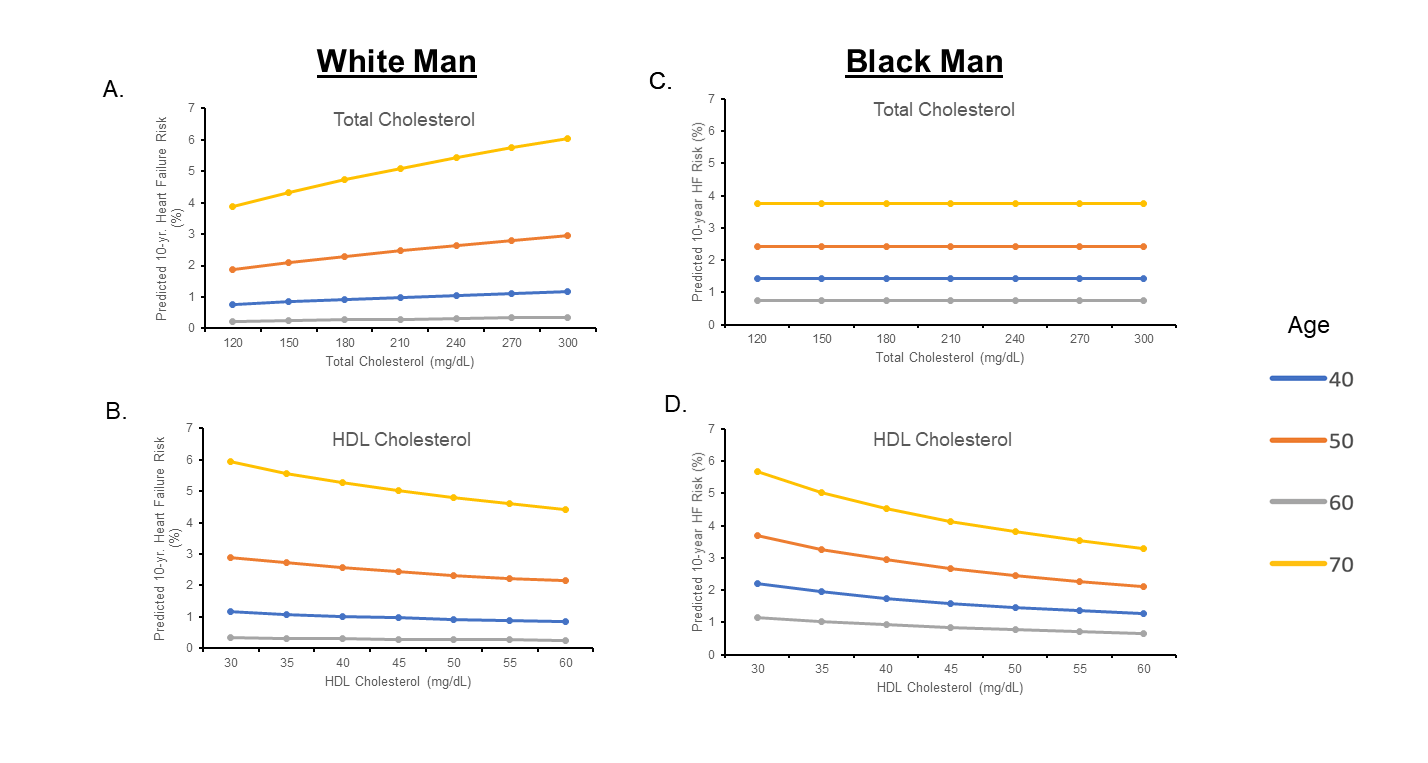

Supplement: S2 Fig — Ten-year predicted risks for heart failure by varying cholesterol levels in a hypothetical White man (A to B) and Black man (C to D) at selected ages, with other risk factors held constant at approximate age-adjusted national means (including nonsmoking). (TIF) [file pone.0240567.s002.tif]

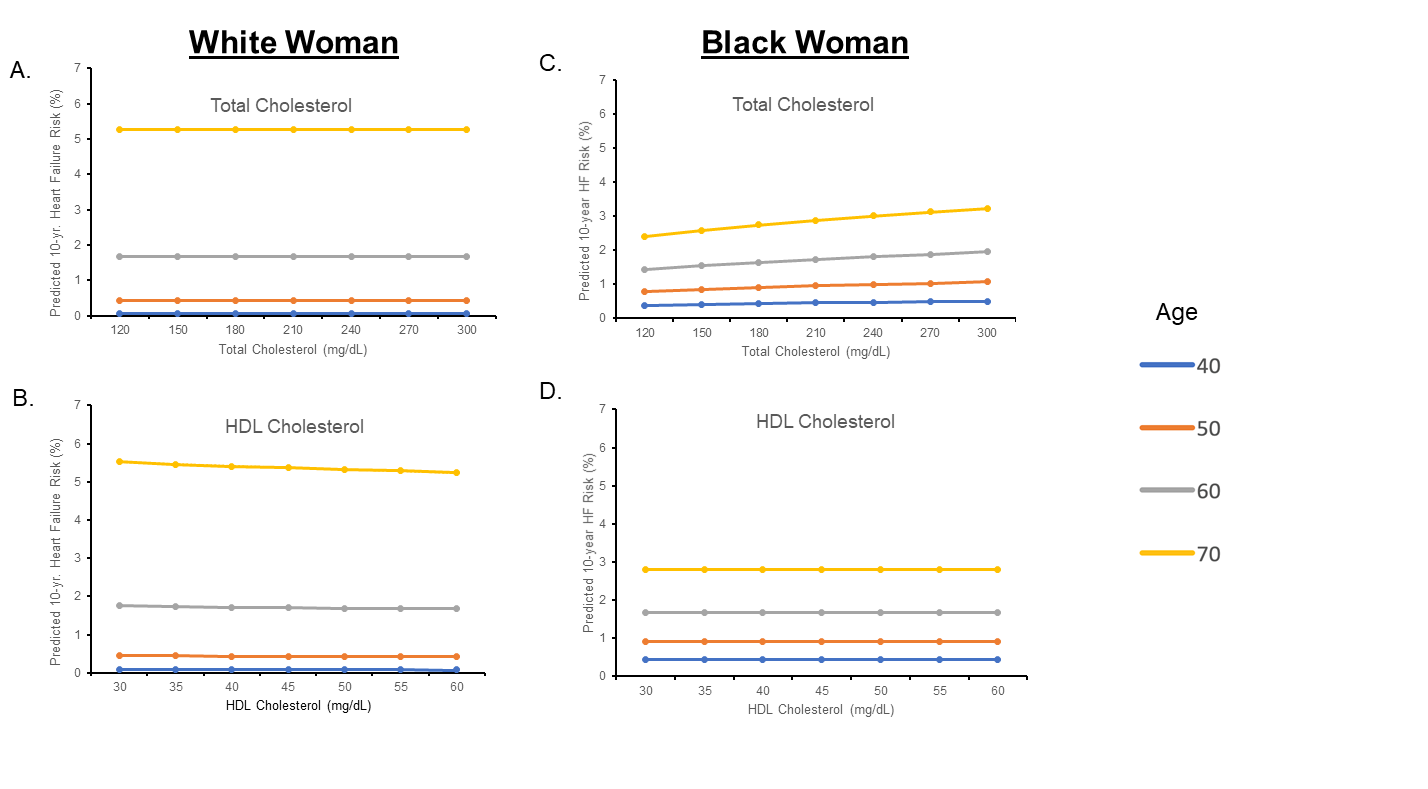

Supplement: S3 Fig — Ten-year predicted risks for heart failure by varying cholesterol levels in a hypothetical White woman (A to B) and Black woman (C to D) at selected ages, with other risk factors held constant at approximate age-adjusted national means (including nonsmoking). (TIF) [file pone.0240567.s003.tif]

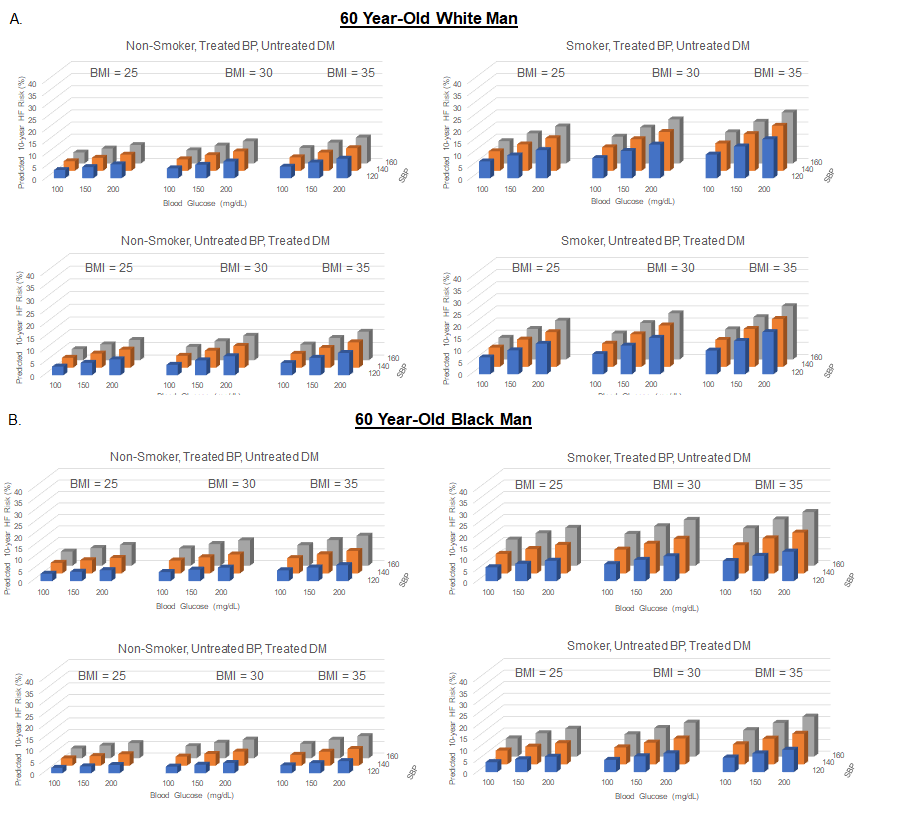

Supplement: S4 Fig — Ten-year predicted risks for heart failure by varying levels of multiple risk factors in a hypothetical White man (A) and Black man (B) at 60 years of age. BMI, body mass index; BP, blood pressure; DM, diabetes mellitus; SBP, systolic blood pressure. (TIF) [file pone.0240567.s004.tif]

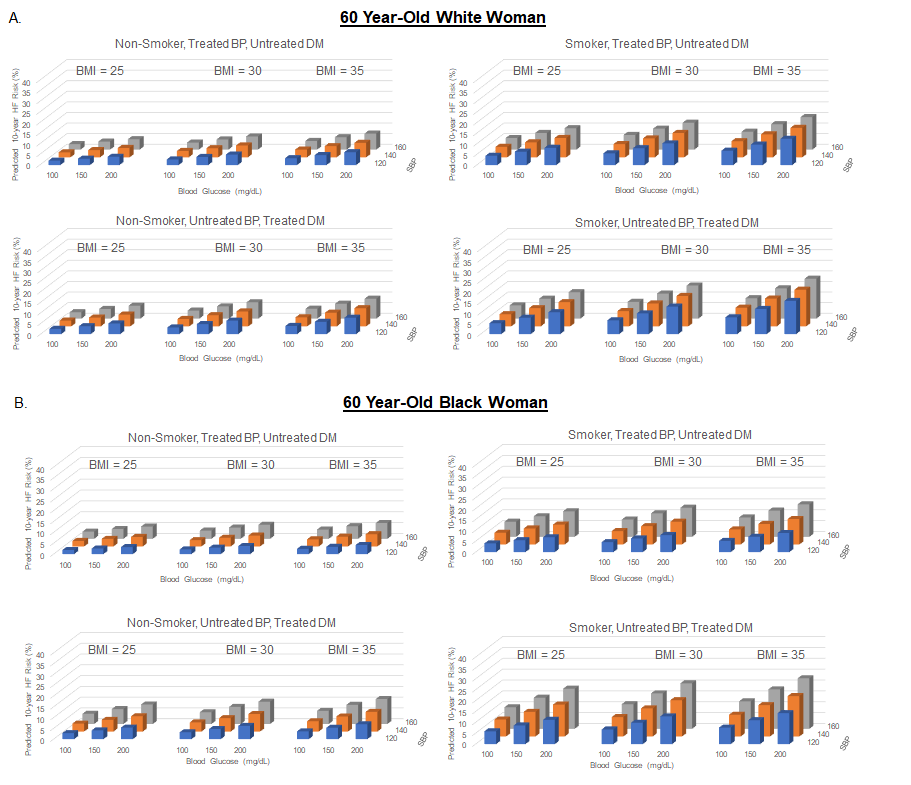

Supplement: S5 Fig — Ten-year predicted risks for heart failure by varying levels of multiple risk factors in a hypothetical White woman (A) and Black woman (B) at 60 years of age. BMI = body mass index, BP = blood pressure, DM = diabetes mellitus, SBP, systolic blood pressure. (TIF) [file pone.0240567.s005.tif]

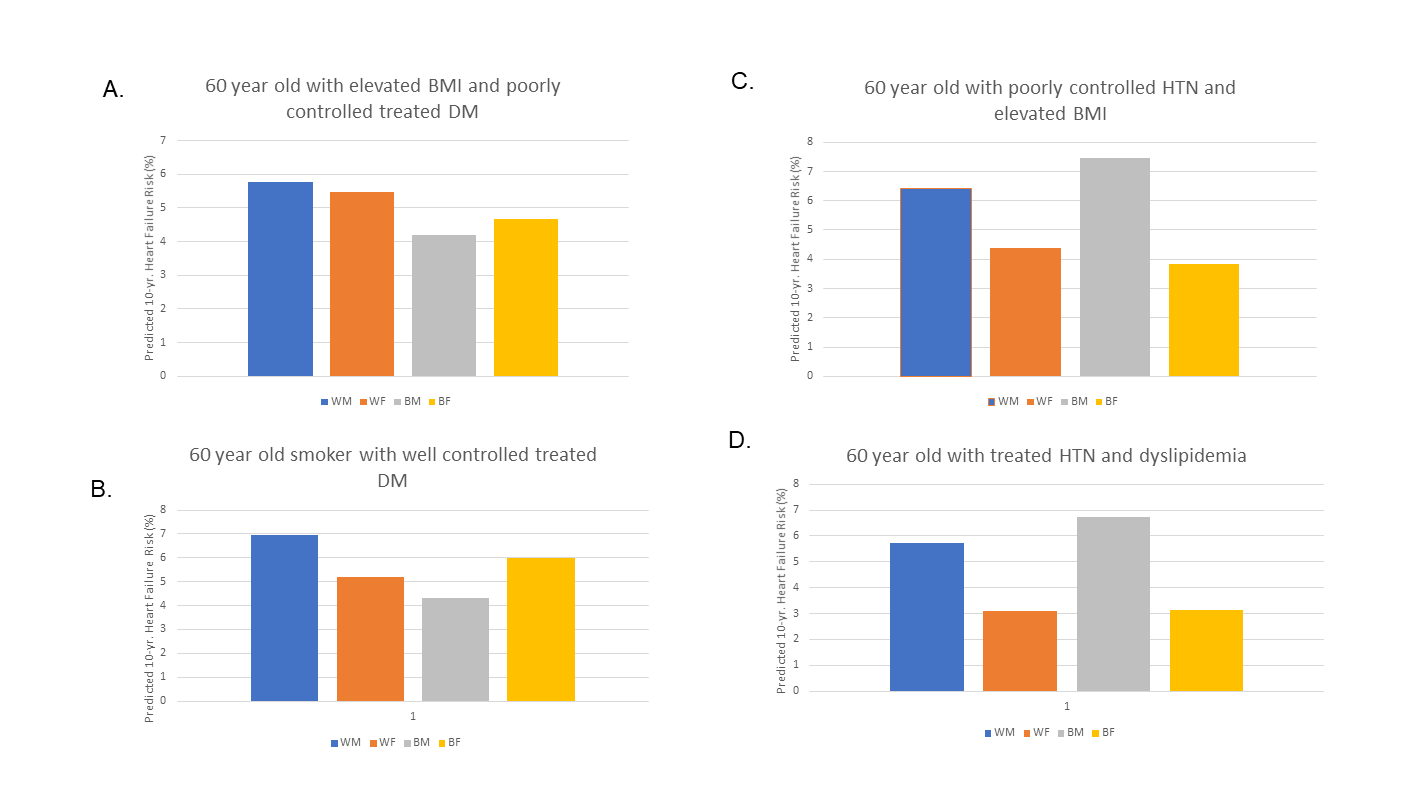

Supplement: S6 Fig — Ten- year predicted risk for heart failure in (A) 60 year old non-smoker with untreated SBP 120 mmHg, treated FG 140 mg/dL, BMI 35 kg/m2, TC = 200mg/dL, HDL-C = 50mg/dL, and QRS 90ms, (B) 60 year old non-smoker with treated SBP 140 mmHg, untreated FG 120 mg/dL, BMI 35 kg/m2, TC = 160 mg/dL, HDL-C = 50mg/dL, and QRS 90ms, (C) 60 year old smoker with untreated SBP 120 mmHg, treated FG 100 mg/dL, BMI 25 kg/m2, TC = 200mg/dL, HDL-C = 50mg/dL, and QRS 90ms and (D) 60 year old non-smoker with treated SBP 140 mmHg untreated FG 100 mg/dL, BMI 30 kg/m2, TC = 240mg/dL, HDL-C = 40mg/dL, and QRS 90ms. BMI = body mass index; DM = diabetes mellitus; HDL = high-density; FG = fasting glucose; LDL-C = lipoprotein cholesterol; SBP = systolic blood pressure; TC = total cholesterol. (TIF) [file pone.0240567.s006.tif]

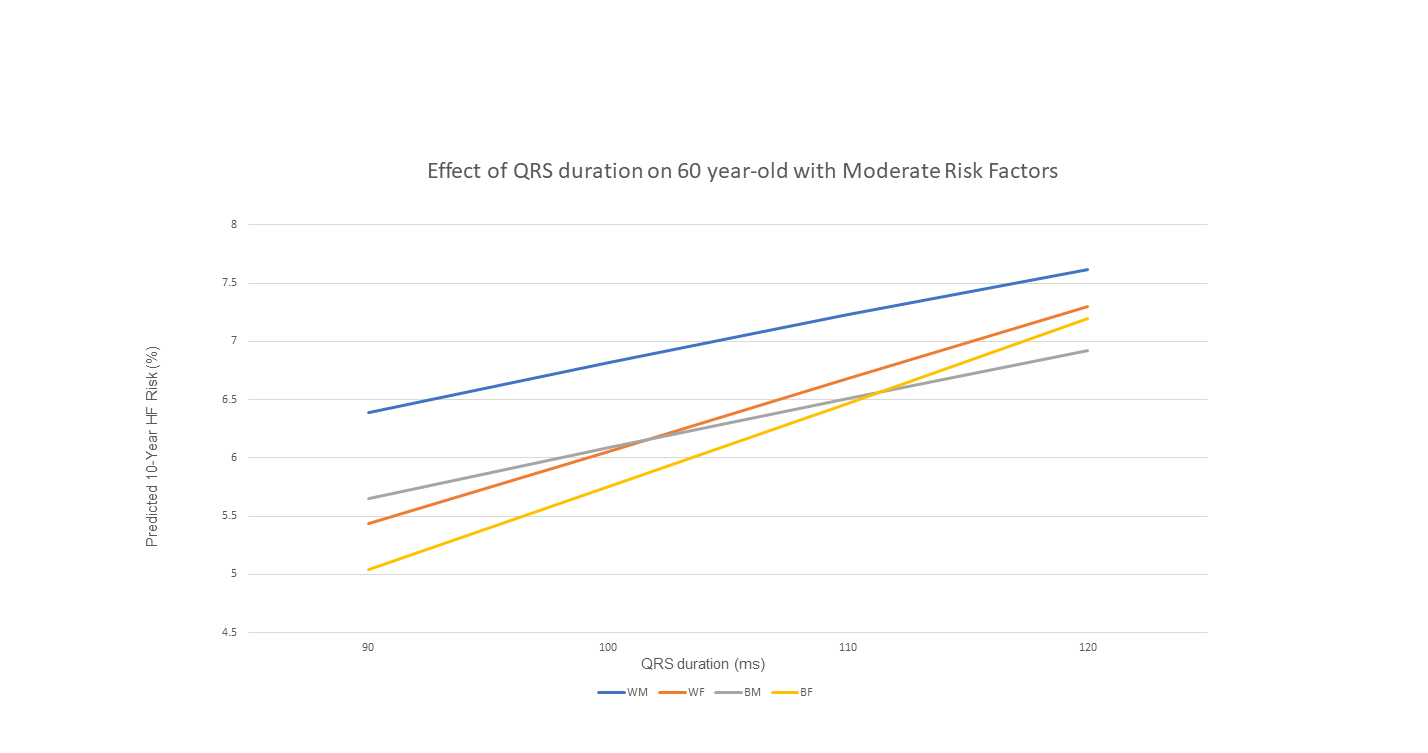

Supplement: S7 Fig — Comparison of ten-year predicted risk for heart failure in a 60 year old of each race-sex group with moderate risk factors with QRS of 90ms or 120ms. Moderate risk factors defined as untreated SBP = 130mmHg; treated FG = 130 mg/dL; BMI = 35kg/ m2; TC = 160 mg/ dL; HDL-C = 40 mg/dL. (TIF) [file pone.0240567.s007.tif]

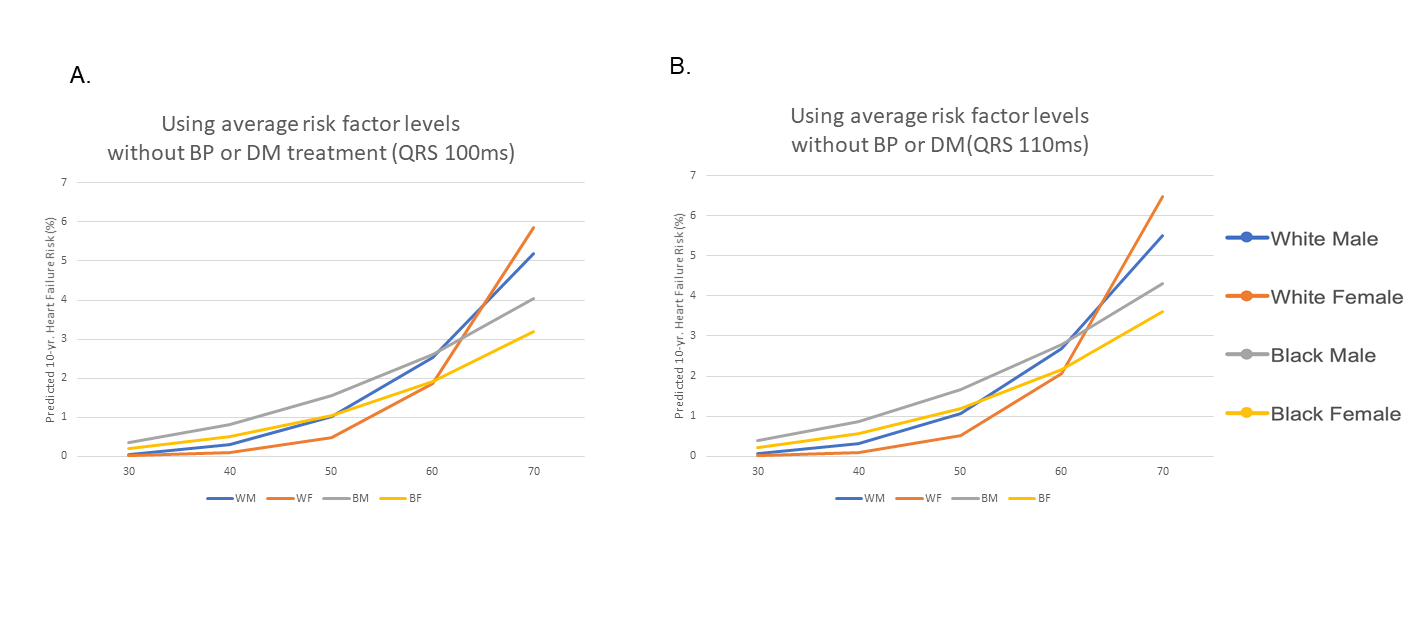

Supplement: S8 Fig — Ten-year predicted risk for heart failure for a hypothetical White and Black man and woman at interval selected ages, with risk factors held constant at approximate age-adjusted national means (among nonsmokers). (A) Ten-year heart failure risk estimates for those not taking antihypertensive or diabetes medications with QRS of 100ms. (B) Ten-year heart failure risk estimates for those not taking antihypertensive or diabetes medications with QRS of 110ms. (TIF) [file pone.0240567.s008.tif]
