# Supplementary material for: Systematic examination of a heart failure risk prediction tool: The pooled cohort equations to prevent heart failure
Source: PLoS One. 2020 Nov 3;15(11):e0240567. doi: 10.1371/journal.pone.0240567 (PMC7608925; doi:10.1371/journal.pone.0240567)
Supplement: S1 Table — (DOCX) [file pone.0240567.s009.docx]

Supplemental Table I: Race and Sex-Specific Coefficients from the Ten-Year Pooled Cohort Equations to Prevent Heart Failure to Calculate Predicted Risk of Heart Failure

|  | **White Men** | **White Women** | **Black Men** | **Black Women** |
| --- | --- | --- | --- | --- |
| **Ln Age (y)** | 41.94 | 20.55 | 2.88 | 51.75 |
| **Ln Age, Squared** | -0.88 | N/A | N/A | N/A |
| **Ln Treated Systolic BP (mm Hg)** | 1.03 | 12.95 | 2.31 | 29.0 |
| **Ln Age×Ln Treated Systolic BP** | N/A | -2.96 | N/A | -6.59 |
| **Ln Untreated Systolic BP (mm Hg)** | 0.91 | 11.86 | 2.17 | 28.18 |
| **Ln Age×Ln Untreated Systolic BP** | N/A | -2.73 | N/A | -6.42 |
| **Current Smoker (1=Yes, 0=No)** | 0.74 | 11.02 | 1.66 | 0.76 |
| **Ln Age×Current Smoker** | N/A | -2.50 | -0.25 | N/A |
| **Ln Treated glucose (mg/dL)** | 0.90 | 1.04 | 0.64 | 0.97 |
| **Ln Untreated glucose (mg/dL)** | 0.78 | 0.91 | 0.58 | 0.80 |
| **Ln Total Cholesterol (mg/dL)** | 0.49 | N/A | N/A | 0.32 |
| **Ln HDL–C (mg/dL)** | -0.44 | -0.07 | -0.81 | N/A |
| **Ln BMI (Kg/m^2^)** | 37.2 | 1.33 | 1.16 | 21.24 |
| **Ln Age× Ln BMI** | -8.83 | N/A | N/A | -5.0 |
| **Ln QRS duration (msec)** | 0.63 | 1.06 | 0.73 | 1.27 |
| **Mean Coefficient× Value *(MeanCV)*** | 171.5 | 99.73 | 28.73 | 233.9 |
| **Baseline Survival (*S_0_*)** | 0.98752 | 0.99348 | 0.98295 | 0.99260 |
| *Calculation of the 10-year risk estimate for HF can best be described as a series of steps: 1) the natural log of age, systolic blood pressure (treated or untreated), glucose (treated or untreated), total cholesterol, HDL-C, body mass index, and QRS duration; 2) calculation of the interaction terms using the natural log of each variable; 3) multiplication of these values by the coefficients from the equation shown above for the appropriate race and sex group; 4) The sum of the coefficientxvalue is calculated for the individual *(IndX)*; **The estimated 10-year risk of a HF event is then formally calculated as:**  $1-{S_{0}}^{e^{(IndX-MeanCV)}}$  Online calculator available at pcphf.org | | | | |

BMI= body mass index; BP= blood pressure; HDL-c= high-density lipoprotein-cholesterol; N/A= not applicable
